# Polarization‐Dependent Elliptical and Rectangular Mie Voids

**DOI:** 10.1002/smll.202511992

**Published:** 2026-02-15

**Authors:** Serkan Arslan, Shaban B. Sulejman, Sebastian Klein, Jonathan Haehner, Julian Schwab, Dominik Ludescher, Lukas Wesemann, Ann Roberts, Harald Giessen, Mario Hentschel

**Affiliations:** ^1^ 4th Physics Institute and Research Center SCoPE University of Stuttgart Pfaffenwaldring 57 70569 Stuttgart Germany; ^2^ ARC Centre of Excellence for Transformative Meta‐Optical Systems School of Physics The University of Melbourne Victoria Australia; ^3^ School of Physics The University of Melbourne Victoria Australia

**Keywords:** metasurface, Mie void, polarization, structural color

## Abstract

Polarization is a key element in the design of nanophotonic devices, such as metasurfaces. It can be used as a parameter to change the performance of an optical device, for example, its transmission spectrum, or in conjunction with tailored anisotropies in nanostructures to control the geometric phase. To design polarization‐sensitive nanophotonic systems, we therefore require anisotropic elements with tailored optical properties. Mie voids, which take the form of lower‐index inclusions in a higher‐index material, have exhibited unique optical properties, such as the confinement of light in air at visible and ultraviolet wavelengths. In this study, we introduce anisotropy to this system to create polarization‐dependent Mie void resonances in elliptical and rectangular voids. We systemically investigate the dependence of the resonances on the geometry of the voids, study the optical mode formation in the system, and demonstrate polarization‐dependent nanoscale color printing. The combination of polarization‐dependent Mie voids and established nanophotonic elements can enable the design of new classes of metasurface designs and advanced polarization‐sensitive photonic devices.

## Graphical abstract

Polarization‐dependent Mie void resonances are realized by introducing anisotropic low‐index inclusions embedded in high‐index materials. Elliptical and rectangular void geometries enable controlled tuning of resonance behavior and optical mode formation. The resulting polarization‐selective response is demonstrated through nanoscale color printing, establishing anisotropic Mie voids as a platform for geometric phase metasurfaces and other polarization‐sensitive nanophotonic devices.

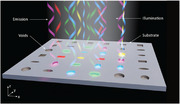

## 1 Introduction

Mie voids, circular holes in the surface of high refractive index dielectric materials, have recently emerged as promising building blocks in the nanophotonic toolbox [1]. In this system, optical resonances occur in the lower‐index region, enabling low‐loss confinement even at ultraviolet wavelengths [1, 2, 3]. These resonators have been applied to nanoscale color printing [1], detecting minute refractive index changes in attoliter‐scale volumes [4], and all‐optically detecting and sizing nanoplastics [5]. In comparison to more widely utilized pillar‐shaped Mie resonators, neither void nor pillar‐based systems can be generally identified as superior; rather, they have individual strengths that should be selected for the given application. Mie voids thus offer additional functionalities and amend the existing pillar designs. To date, the study of Mie voids has been limited to circular cross‐sections, forming cylindrical volumes that approximate the symmetry and modal characteristics of classical solid Mie resonators. Due to in‐plane rotational symmetry, previously investigated Mie void resonances have been polarization independent. However, anisotropic “meta‐atoms” are required for many polarization‐dependent nanophotonic applications, such as geometric phase (Pancharatnam–Berry) metasurfaces [6, 7, 8], polarization‐dependent color printing [9, 10, 11], color filtering [12, 13, 14], and bio‐sensing [15, 16, 17, 18, 19].

While polarization‐dependent dielectric Mie resonators have already been widely explored [20, 21, 22, 23], here we aim to introduce this previously unexplored degree of freedom into the Mie void platform, adding to its inherent advantages such as material dispersionless and lossless resonances in air, and an easily accessible mode volume. To this end, we fabricate Mie void metasurfaces with elliptical and rectangular cross‐sections in gallium arsenide (GaAs) substrates. We experimentally investigate the polarization dependence of the resulting optical resonances using white‐light microspectroscopy. Furthermore, we computationally investigate the optical mode formation (up to third‐order) via full‐wave electromagnetic simulations. We systematically study variations in the size, aspect ratio and depth of the voids and their associated impact on the optical response. Lastly, we experimentally demonstrate their capacity to produce polarization‐dependent colors for color switching and nanoscale color printing. Overall, this work highlights the polarization degree of freedom in Mie void metasurface designs and contributes to the broader field of structural coloration [24, 25, 26, 27, 28, 29, 30, 31, 32, 33, 34].

## 2 Results and Discussion

**Figure 1:**
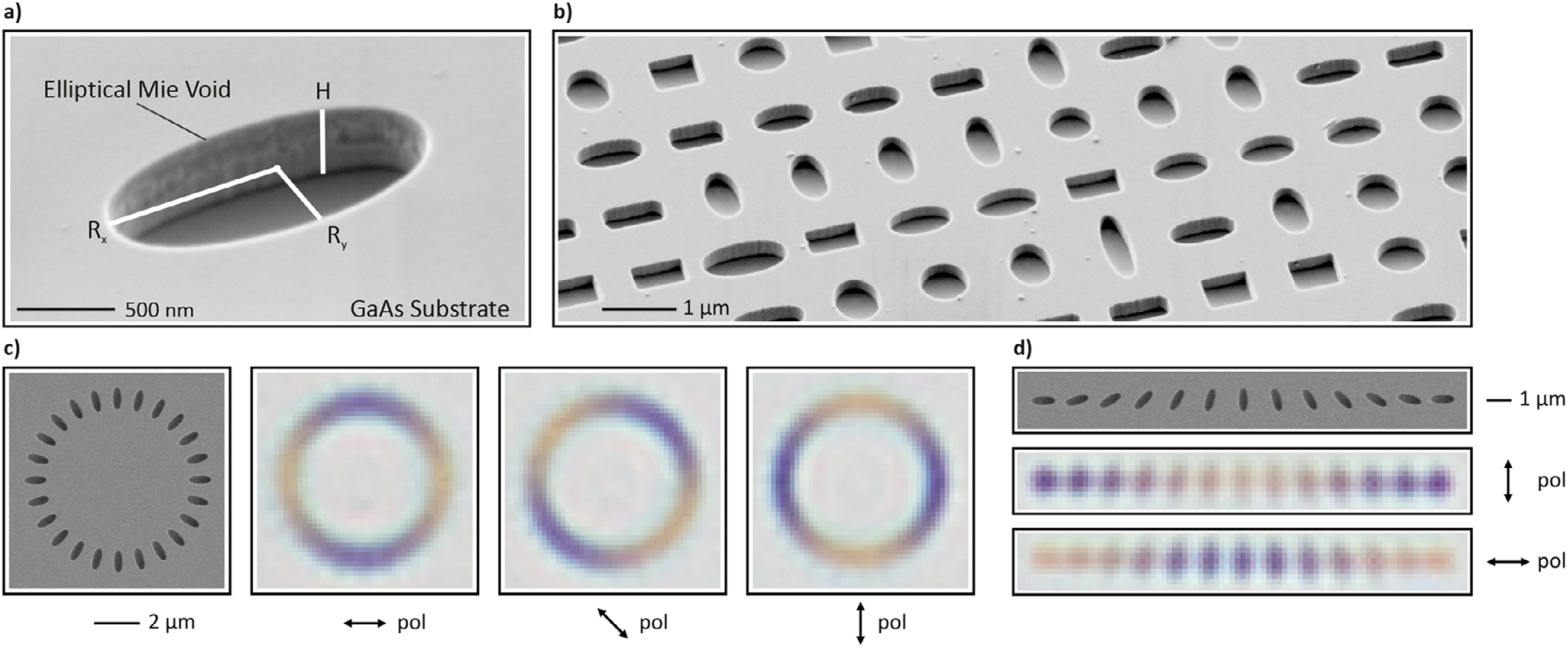
SEM and microscope images of fabricated Mie void arrays. (a) Tilted‐view SEM image of a single elliptical Mie void in a GaAs substrate. (b) Tilted‐view SEM image of a Mie void metasurface consisting of elliptical and rectangular Mie voids with various sizes and aspect ratios. (c) SEM image (left) of a circular array of identical elliptical voids, and corresponding bright‐field microscope images (right). (d) SEM image of a linear arrangement of elliptical voids rotated in 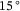 increments (top). The corresponding microscope images under vertical and horizontal polarization confirm that each void color is individually governed by its orientation relative to the polarization axis (bottom). We note that the apparent blurriness of the optical microscope images is related to the nanoscale dimensions of the imaged Mie voids.

The tilted‐view scanning electron microscope (SEM) image shown in Figure 1a presents an elliptical Mie void etched into the surface of a GaAs wafer. The presented concepts are in principle transferable to any other high‐refractive‐index dielectric material such as Si, SiC, Ge, GaP, diamond, or even Van der Waals heterostructures [1, 3, 35, 36]. We define the long and short axes of an elliptical void as the x and y axes, respectively, with corresponding lengths of the semi‐axes given by $R_{x}$ and $R_{y}$. The void has a depth of H. Figure 1b highlights the fabrication of elliptical voids of various sizes and aspect ratios. Our fabrication method is based on standard electron beam lithography (EBL) and reactive ion etching (RIE)‐based dry etching (details can be found in the Methods section). Figure 1c,d demonstrates the polarization dependence of the optical resonances in a visual approach. In panel c, we arranged identical elliptical Mie voids with the dimensions $R_{x}=490\;nm$, $R_{y}=200\;nm$, and H=300nm in a circle and imaged them with a white‐light microscope equipped with a 20× objective. When the polarization is aligned with, or perpendicular to, the long axis of the ellipse, the reflected color appears yellow or blue, respectively. Rotating the polarizer causes the colors to cycle about the circular array, as illustrated in the corresponding microscope images. Notably, the observed colors arise from individual voids rather than from collective effects. To reinforce this point, we fabricated identical Mie voids arranged linearly, with each ellipse incrementally rotated by 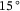, as depicted in Figure 1d. From the associated microscope images under linear polarization, it is evident that the color of each void is determined by its orientation relative to the polarization. Under perpendicular polarization, the 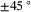
‐oriented ellipses display identical pinkish hues. The same color is observed under unpolarized illumination for all ellipses, independent of their orientation, representing the average of the two orthogonal polarization states.

**Figure 2:**
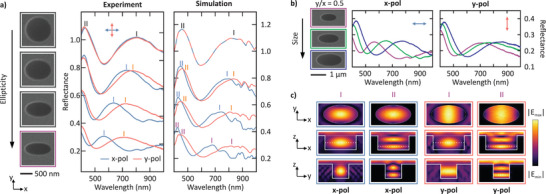
Polarization‐dependent spectral response of elliptical Mie voids. (a) SEM images (left) of Mie voids with increasing ellipticity ($R_{x}=530\;nm$ and $R_{y}=530$, 425, 330 and 270nm) (top to bottom). Center: Measured reflectance spectra under x‐ (blue curves) and y‐polarization (orange curves) in a waterfall plot (0.25 vertical offset, respectively). (b) SEM images (left) of elliptical Mie voids with a fixed aspect ratio ($R_{y}/R_{x}=0.5$) and increasing size. Middle and right: Corresponding experimental (middle) and simulated (right) reflectance spectra for x‐ and y‐polarization. (c) Simulated electric field strength distributions of the two lowest‐order resonances for x‐ and y‐polarized light in the smallest void from (b) (purple frame), shown in the xy‐, xz‐ and yz‐planes.

We quantitatively investigated the polarization dependence of elliptical voids by measuring the white light reflectance spectra (see Methods for details). We fabricated arrays of Mie voids with a periodicity of 1200nm and representative examples are presented in the SEM images in Figure 2a (left). First, we consider a circular void of radius $R_{x}=R_{y}=530\;nm$ and depth H=480nm, before subsequently increasing the ellipticity by holding $R_{x}$ fixed as the long axis and reducing $R_{y}$ to 425nm, 330nm and 270nm (top to bottom in Figure 2a‐left). The center panel of Figure 2a shows the measured reflectance spectra over a wavelength range 400–1000nm for x‐polarization (blue curve) and y‐polarization (orange curve). The spectra are presented in a waterfall plot with a vertical offset of 0.25 per spectrum.

In the case of the circular void (top of Figure 2a), the reflectance spectra for x‐ and y‐polarization are approximately identical, as expected due to the in‐plane rotational symmetry. The small deviations can be attributed to slight anisotropy arising from fabrication errors or minor misalignment in the experiment. The spectra of the circular voids exhibit two peaks in the measured range, corresponding to the two lowest‐order resonances labeled I and II in the figure. As we reduce $R_{y}$ and therefore increase the ellipticity of the voids, one can observe a splitting of the lowest order resonance I between the two polarization states that strengthens with an increasing ellipticity. Both peaks for x‐ and y‐polarization undergo a blueshift relative to the circular void as $R_{y}$ decreases. This can be attributed to a decreased total mode volume of the void. Notably, the spectra for x‐polarization (blue curve) parallel to the fixed axis $R_{x}$ underwent a stronger blueshift compared to the spectra for polarization along the y‐axis, which is the axis being reduced. The spectral location of the fundamental mode is mostly determined by the width of the void along a direction perpendicular to the polarization, while changes in the width of the void parallel to the polarization lead to smaller spectral shifts. This behavior is analogous to the behavior in rectangular cavity waveguides for the ${\text{TE}}_{01}$ and ${\text{TE}}_{10}$ modes, where the waveguide dispersion depends on the dimension orthogonal to the polarization. The evolution of the spectra also reveals a reduced amplitude of the reflectance for an increasing ellipticity. This is due to a reduced volume of the structure and hence a decreased oscillation strength of the resonance.

The experimental results are confirmed by full‐wave simulations of the Mie void structures using a commercial electromagnetic solver (COMSOL Multiphysics 6.1, see Methods for details). The resulting simulated reflectance spectra are compared to the experimental results in Figure 2a (right). As before, the reflectance spectrum was polarization independent in the case of the circular void, and the splitting and blueshift observed in the experiment are also reproduced. Deviations in the spectral positions between simulated and experimental reflectance peaks can, for example, be attributed to uncertainties in the extracted void dimensions from SEM images. Additionally, the simulated voids were modeled as cylindrical volumes, whereas in the experiment, the etching process can produce non‐vertical sidewalls, leading to truncated conical voids and a generally smaller volume than the corresponding ideal cylinders. Consequently, the simulated spectra are generally redshifted compared to the experimental spectra. The spectral features above 700nm are likely due to in‐plane lattice modes in the simulated array, which are not observed experimentally.

Figure 2b examines the optical response of elliptical Mie voids under a size variation for a fixed aspect ratio of approximately $R_{y}/R_{x}=0.5$. The axes of the voids in the SEM images (top to bottom) are $R_{x}=520\;nm$, 680nm and 840nm (along the x‐axis) and $R_{y}=270\;nm$, 320nm and 440nm (along the y‐axis). The reflectance spectra for each case are presented for x‐polarization (Figure 2b‐middle) and y‐polarization (Figure 2b‐right). As expected and observed for circular Mie voids [1], the spectra for each polarization experience a redshift for an increasing void size. As a result, it enables tuning of the resonances across a broad spectral range, facilitating their use in a wide variety of nanophotonic applications.

For the smallest void (purple frame in Figure 2b), Figure 2c depicts the simulated electric field strength distribution across the three spatial planes for the two lowest‐energy resonances (at the peak positions of the reflectance) under x‐ and y‐polarized illumination, respectively. Slices through the two vertical planes passing through the center of the void were obtained (xz and yz), while the xy‐plane was set to coincide with the location of maximum field strength, as indicated by the white dotted line. Generally, the observed field distributions resemble those previously reported for circular voids in Ref. [1]. The field profiles in the xy‐plane demonstrate that, for both the first and second‐order resonances, the mode aligns with the incident polarization direction. Vertical cross‐sections in the xz‐ and yz‐planes enable an identification of the mode order, as either one or two field nodes are observed along the axial direction.

**Figure 3:**
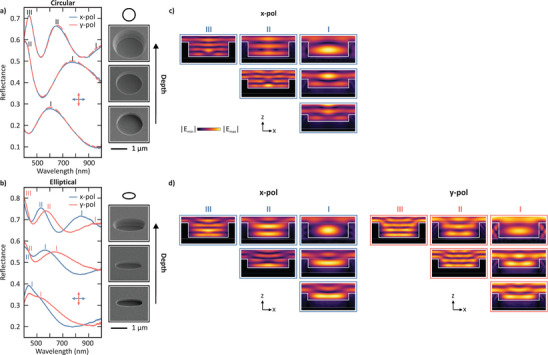
Polarization‐dependent optical response of elliptical Mie voids under depth variation. Depth‐dependent reflectance spectra for circular (a) and elliptical (b) Mie voids with a fixed in‐plane size and linearly polarized illumination. In each case, three depths (bottom to top: 290, 380 and 630nm) were fabricated, as confirmed in the SEM images. The circular voids have a radius $R_{x}=R_{y}870\;nm$. The elliptical voids have radii $R_{x}=870\;nm$ and $R_{y}=350\;nm$. Spectra are presented as waterfall plots with a 0.25 vertical offset. Increasing the void depth leads to a progressive redshift of the fundamental resonance (mode I) and the emergence of higher‐order modes (II and III). (c) Electric field strength distributions for each void in (a) at the resonant wavelengths in the xz‐plane and x‐polarization. Higher‐order modes form Fabry–Pérot‐type electric field distributions along the depth of the void. (d) Electric field strength distributions for each void in (b) at the resonant wavelengths in the xz‐plane for x‐ and y‐polarization.

Next, we systematically investigated the influence of the void depth on the optical response for circular (Figure 3a) and elliptical (Figure 3b) voids. Voids were fabricated with depths of 290, 380 and 630nm, as illustrated in the respective SEM images. The radius $R_{x}$ was fixed at 870nm for all voids, while $R_{y}$ was fixed at 350nm for the elliptical voids. The slight increase of the in‐plane void radii observed for larger etching depths originates from the etching process and does not affect the qualitative interpretation or the conclusions drawn from the subsequent analysis. As in Figure 2a, the reflectance spectra in Figure 3a,b are presented as waterfall plots with a vertical offset of 0.25. The shallowest circular void exhibits a single resonance peak near 600nm, associated with the fundamental resonance, labeled I. As the depth increases, this resonance redshifts to approximately 750nm and a second‐order peak (labeled II) emerges in the visible spectral range at 400nm. For the deepest void, the fundamental mode shifts further to wavelengths beyond the measured range (above 1000nm), while a third‐order peak appears above 400nm. In addition to the in‐plane dimensions, the void depth also serves as a tuning parameter, offering broad flexibility in designing desired spectral responses. Overall, the dependence of the mode formation on the void depth can be understood as follows: For an increasing mode number, we observe an increasing number of electric field strength maxima and minima in the vertical direction, reminiscent of standing‐wave or Fabry–Perot type resonances. Only in case of the largest diameter void, we observe up to three electric field strength maxima. These modes can be independently tuned from the in‐plane modes, for which modes of increasing order have also been observed.

Similar results are observed in the case of the depth sweep for the elliptical voids. The key difference is a splitting of the spectra under x‐ and y‐polarization, which behave similarly as described above. For circular voids, the mode I is observed for the shallowest void, while the modes II and III subsequently appear for increasing depths. The spectral positions of the reflectance peaks are blue‐shifted relative to those of the circular voids with the same depth due to a smaller mode volume of the void (see Figure 2a). Again, we can identify up to three electric field strength maxima in the vertical direction for the highest order mode, which holds for both incident polarizations.

**Figure 4:**
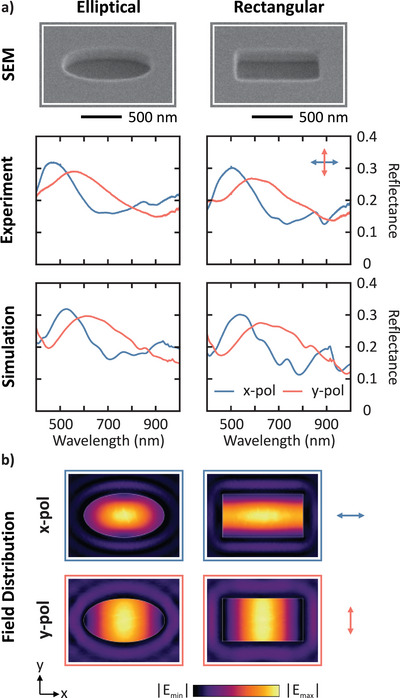
Comparison of elliptical and rectangular Mie voids. (a) Experimental and simulated polarization‐dependent reflectance spectra of elliptical (left) and rectangular (right) voids with matched lateral dimensions. Both geometries exhibit similar polarization splitting of the fundamental mode. (b) Simulated electric field strength distributions in the xy‐plane at half the void depth. The modes align along the x‐ and y‐axes of the respective void, parallel to the incident polarization.

The primary motivation for employing elliptical Mie voids lies in their ability to support polarization‐dependent resonances, which necessitate structural anisotropy. Among the possible anisotropic shapes, the ellipse constitutes a natural deformation of the circular void considered in Reference [1]. In the broader literature on dielectric Mie resonators, circular and elliptical resonators, as well as square and rectangular geometries, are also utilized [37, 38, 39, 40]. Therefore, we also compare an elliptical void (top left of Figure 4a) with a rectangular void (top right of Figure 4a). The semi‐axes of the elliptical voids were approximately $R_{x}=440\;nm$ and $R_{y}=240\;nm$. On the other hand, the side lengths of the rectangular void were chosen to match the diameters of the elliptical voids, i.e. $L_{x}=880\;nm$ and $L_{y}=480\;nm$, where $L_{x}$ and $L_{y}$ are the side lengths along the x and y axes, respectively. Both the elliptical and rectangular voids had a depth of 340nm.

The experimental and simulated polarization‐dependent reflectance spectra are presented for the elliptical (left) and rectangular (right) voids in Figure 4a. A comparison of the experimental data reveals that rectangular voids exhibit spectral responses similar to those of elliptical voids. As before, there is a polarization splitting of the fundamental mode. The spectral positions of the resonances for rectangular voids are slightly redshifted compared to those of the ellipses, likely due to a slightly larger volume. This redshift is also observed in the simulation. The simulations further reveal stronger modal features from in‐plane grating modes for the rectangular voids, particularly under x‐polarized illumination, with a prominent feature at 900nm also visible in the experimental spectrum near 850nm. For applications requiring the suppression of these modes, elliptical geometries and aperiodic or disordered arrangements may be preferred [41, 42, 43].

Figure 4b shows the simulated electric field strength distributions for both the elliptical and rectangular voids. The field distribution was extracted in the xy‐plane, located at half the void depth, which corresponds to the maximum of the vertical field strength. The simulations reveal similar mode behavior for the rectangular and elliptical voids. In both cases, the polarization of the illumination determines the orientation of the mode. Note that elliptical and rectangular voids are both equally suited for applications. One could have expected the rectangular voids to exhibit weakened performance, as Mie resonances are often associated with mirror cavities, which demonstrate superior performance in spherical or elliptical geometries. However, this was not the case here, and both classes of voids lead to resonant modes. Additional experimental data of the reflectance spectra spanning a broad parameter space of circular, elliptical, square and rectangular voids are presented in the Figures S1–S10.

**Figure 5:**
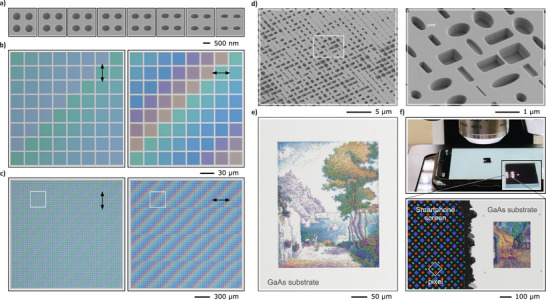
Nanoscale color printing using elliptical and rectangular Mie voids. (a) SEM images of square arrays with an increasing void ellipticity. (b) Bright‐field microscope images of the same arrays under vertical (left) and horizontal (right) polarization. (c) Wide‐field microscope image of the patterned area (2mm edge length). (d) SEM overview and magnified view of a color print employing elliptical and rectangular Mie voids with four different depths for an expanded color gamut. (e) Microscope image of a nanoprinted, 320μm × 400μm reproduction of *Capo di Noli* (1898) by Paul Signac (void period of 1200nm). Reproduced with permission (Photocredit “Capo di Noli” by “Paul Signac”, photocopyright “Wallraf–Richartz Museum & Fondation Corboud, Köln, Germany”). (f) Top: GaAs sample on a smartphone screen, showing six illuminated color prints under a microscope. Bottom: Microscope image of a second nanoprint ‐ *Café Terrace at Night* (1888) by Vincent van Gogh. Reproduced with permission (Photocredit “Café Terrace at Night” by “Vincent van Gogh”, photocopyright “Kröller Müller Museum, Otterlo, Netherlands”). The OLED pixels of the smartphone (458 dots per inch) are resolved next to the metasurface (21 167 dots per inch) for scale.

The vibrant colors of Mie voids were previously reported in Ref. [1]. Compared to the circular voids presented there, elliptical Mie voids, as demonstrated in this work, offer the additional benefit of polarization‐dependent optical properties, which lead to polarization‐sensitive structural coloration (Figure 1c,d). This is explored in Figure 5. To this end, square arrays of eight different void geometries, starting from circular to an increasing ellipticity, were fabricated, as shown in the SEM images in Figure 5a. Figure 5b displays microscope images of the arrays under vertical (left) and horizontal (right) polarization. The SEM images correspond in order of the arrays in the bottom row of the microscope images, with circular voids on the far left and the most elliptical voids on the far right. In the microscope image, each row is shifted one array position to the right relative to the row below it. Under vertical polarization (along the short axis of the ellipses), only subtle changes in reflected colors are observed, with hues transitioning from turquoise to blue. On the other hand, under horizontal polarization (along the long axis of the ellipses), the colors change significantly, ranging from turquoise over blue, violet to orange in a cyclic fashion. This observation is consistent with the results presented in Figure 2a, where the resonances for the polarization along the long axis exhibited a stronger blueshift compared to those along the short axis for an equivalent change from circular to elliptical voids. Figure 5c depicts the same sample under a wider field of view, covering a patterned area of 2mm × 2mm. Such a large‐area and polarization‐dependent color switching may be useful for applications including anti‐counterfeiting and security tagging [44].

A key factor in achieving a wide color gamut and high saturation range is the variation of the in‐plane parameters and the depth. This was previously realized using focused ion beam (FIB) milling that enabled grayscale control of individual void depths within a single fabrication step. Despite its advantages, this method is limited by the relatively slow writing speed of the FIB process, which restricts the achievable structured area. In contrast, the EBL process used in this work, combined with subsequent etching, enables large‐area structuring and higher throughput fabrication. Although grayscale depth control is not possible with this method, multiple depths within a single sample can be realized through a multilayer fabrication approach. To maximize the achievable color gamut, the color prints were optimized for viewing under linear polarization, introducing an additional degree of freedom in the color tuning via rotation of the individual voids. Although elliptical and rectangular voids are similar in their optical response, both are employed here to enhance the gamut and saturation.

Figure 5d presents a wide field of view SEM image and a magnified view of such a color print. The image reveals the use of elliptical and rectangular voids of various aspect ratios and sizes. Notably, the voids are oriented along both the vertical and horizontal axes of the color print. Closer inspection of the magnified region confirms that four void depths were implemented using a multilayer fabrication process. Generally, deeper voids yield darker and more saturated colors, while shallower voids produce brighter hues. Figure 5e shows an optical microscope image of a nanofabricated reproduction of the 1898 painting *Capo di Noli* by French painter Paul Signac. The nanoprint measures 320μm × 400μm in size, with a void period of 1200nm. Figure 5f illustrates the various length scales involved in the color print. The top panel depicts a Mie void metasurface sample positioned on a smartphone screen underneath a microscope. The magnified view of the sample reveals six small, bright rectangles illuminated by the microscope, each corresponding to an individual color print as shown in Figure 5e. This highlights the scalability of the method, as the full sample contains 18 color prints fabricated on a single substrate. The bottom panel provides a microscope image of one of the color prints located near the edge of the substrate. This structure reproduces the 1888 painting *Café Terrace at Night* by Vincent van Gogh. Compared to the subtler hues in the color print in Figure 5e, this reproduction exhibits a greater saturation in the colors. For reference, the left side of the image shows individual OLED subpixels from the underlying smartphone display. The image is a two‐focal‐plane stack, necessitated by the height difference between the GaAs substrate and the screen, which exceeds the depth of field of the microscope. The pixel size of the smartphone is 55.5μm, corresponding to a resolution of 458 dots per inch. On the other hand, the pixel resolution of the metasurface reached a value of 21 167 dots per inch. Further examples of five color prints are presented in the Figure S11. The CIE coverage of the color palette utilized for the color prints is displayed in Figure S12. Compared to common structural colors, which often utilize a combination of localized and delocalized grating resonances, our Q factors are lower and hence the resulting colors less saturated since, their spectral range is broader.

### 2.1 Summary

In conclusion, we have extended the concept of Mie voids beyond their original circular geometry by introducing anisotropic elliptical and rectangular cross‐sections, thereby enabling polarization‐dependent resonances. Using a combination of full‐wave electromagnetic simulations and experimental white‐light microspectroscopy, we demonstrated that the in‐plane dimensions and void depth are both critical in defining the spectral position, polarization splitting, and modal behavior of the resonances. Our results revealed that the spectral position of the resonances is strongly influenced by the void dimension perpendicular to the polarization, while the void depth served as an independent tuning parameter. The combined variation of axial and lateral geometry enables fine control over the optical response of individual voids. We demonstrated the application of this tunability to polarization‐switchable color generation and high‐resolution nanoscale color printing, achieving structural coloration at densities exceeding 21 000 dots per inch. Utilizing multilayer fabrication, we realized multiple depths on a single substrate, producing vivid color prints. These capabilities highlight the potential of anisotropic Mie voids for applications in geometric phase metasurfaces, polarization‐resolved biosensing, and large‐area security features. Our work establishes Mie voids as a versatile, lowloss platform for air‐mode dielectric nanophotonics, with independent access to all three geometric parameters (width, height and aspect ratio) for complete control over polarization‐sensitive resonance engineering.

### 2.2 Methods

#### 2.2.1 Fabrication

A positive‐tone electron‐beam resist (AR‐P 6200.13, 400nm, Allresist) was spin‐coated on pre‐cleaned GaAs substrates (2000rpm for 5sec, followed by 4000rpm for SI55sec, soft bake at 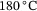 for 3min). Electron beam lithography was performed on a commercial system (VOYAGER, Raith GmbH, 50kV acceleration voltage, 60μm aperture, and 10nm step size, area dose of $200\;\mu C/\;{\mathrm{cm}}^{2}$). After exposure, the resist was developed in AR 600–546 for 90sec, followed by immersion in AR 600‐60 stopper solution for 30sec. Dry etching was performed by using an inductively coupled plasma system (Oxford Instruments Plasmalab System 100, 60W radio frequency power, 350W inductively coupled plasma power, 6sccm SiCl4 flow rate, 5sccm helium flow rate and a chamber temperature of 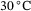, resulting etching rate about 12.$6\;{\mathrm{nms}}^{-1}$). Once etching had completed, the resist mask was removed by briefly immersing the sample in N‐ethylpentedrone to ensure clean mask removal. Finally, the sample was rinsed with acetone and isopropyl alcohol to eliminate any residual contaminants, completing the fabrication process [5].

#### 2.2.2 Structural Characterization

The in‐plane dimensions of individual fabricated Mie voids are extracted from normal‐view SEM images, while the void depths are determined from SEM images acquired at tilt angles between 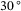 and 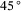.

#### 2.2.3 Microspectroscopy

Reflectance spectra of ensembles of Mie voids were recorded based on the method established in Ref. [1], with an additional motorized linear polarizer positioned in the collection path of the microscope. We use a custom‐built microspectroscopy configuration based on an inverted microscope (Nikon TE2000‐U) and a Princeton Instruments grating spectrometer (SP2500i) with a Peltier‐cooled CCD camera (PIXIS 256E). The sample was placed on a motorized XY stage (Märzhäuser) and illuminated by a fibre‐coupled laser‐driven white light source (Energetiq EQ‐99XFC). A high‐magnification objective (Nikon TU Plan Fluor ELWD 60x, NA 0.7) was used to observe the void arrays. The light source was projected onto the sample via a custom‐built Köhler illumination path, which was designed to illuminate only a small range of incident angles, i.e. near‐normal incidence. A motorized linear polarizer selected the desired polarization. The microscope was connected to the entrance slit of the spectrometer via a modified 4f configuration, which contained an aperture in the microscope image plane (field stop), as well as in the intermediate Fourier plane (aperture stop). Because the sample reflectance can be near‐zero, both of the apertures were closed to block any light scattered by the substrate, as well as light scattered/diffracted into larger angles. In this way, only the zeroth‐order reflectance is measured, which can be directly compared to the numerical calculations. The reflectance was extracted by normalizing the measured reflected signal of a void ensemble to that of the bare GaAs substrate and multiplying by the known reflectance spectrum of GaAs [45].

#### 2.2.4 Simulations

Numerical simulations were performed using COMSOL Multiphysics 6.1 implementing the finite element method within the wave optics module. Periodic Floquet boundary conditions were applied to the sides of a rectangular unit cell of the metasurface to model an infinite 2D square lattice. The unit cell was taken to be 1200nm thick. The air superstrate and void were assumed to be homogeneous and lossless materials with a refractive index of 1. The gallium arsenide was modeled using ellipsometry data from Ref. [46], which incorporated losses. Port boundary conditions were applied to the top and bottom of the unit cell to launch and absorb electromagnetic plane waves, respectively, and to monitor the amplitude and phase of reflected and transmitted orders. The resultant calculations were normalized to those of the incident field. Due to the high refractive index of gallium arsenide, perfectly matched layers were also applied above the top port and below the bottom port to absorb outgoing electromagnetic waves, with material properties matching the superstrate and substrate, respectively. The perfectly matched layers had a thickness of 250nm, corresponding to a quarter of the maximum wavelength considered in the simulations (1000nm). The finite mesh elements in the model had a maximum size of 100nm, corresponding to a quarter of the minimum wavelength considered in the simulations (400nm), and a minimum size of 5nm. A swept quadrilateral meshing distribution with five rows of swept elements was used for the perfectly matched layers. It applied a rational coordinate stretching type suitable for waves propagating at various incident angles, with a scaling factor of 1.5 and scaling curvature of 0.5.

## Funding

This work was supported by the Ministerium für Wissenschaft, Forschung und Kunst Baden–Württemberg (RiSC Project “Mie Voids”, ZAQuant), Vector Stiftung MINT‐Innovationen, Carl‐Zeiss‐Stiftung (CZS Center QPhoton), Baden–Württemberg–Stiftung (Opterial), European Research Council (ERC Advanced Grant Complexplas & ERC PoC Grant 3DPrintedOptics), Bundesministerium für Bildung und Forschung, Deutsche Forschungsgemeinschaft (SPP1839 “Tailored Disorder” and GRK2642 “Towards Graduate Experts in Photonic Quantum Technologies”), and the Australian Government through the Australian Research Council (ARC) Centre of Excellence grant CE200100010. S.B.S acknowledges the support of the Ernst and Grace Matthaei Scholarship, the Australian Government Research Training Program Scholarship and the Dr Albert Shimmins Fund.

## Conflicts of Interest

The authors declare no conflicts of interest.

## Supporting information


**Supporting File 1**: smll72788‐sup‐0001‐SuppMat‐part1.pdf.

**Supporting File 2**: smll72788‐sup‐0002‐SuppMat‐part2.pdf.

## Data Availability

The data that support the findings of this study are available from the corresponding author upon reasonable request.
