# Supplementary material for: Polarization‐Dependent Elliptical and Rectangular Mie Voids
Source: Small. 2026 Feb 15;22(21):e11992. doi: 10.1002/smll.202511992 (PMC13081118; doi:10.1002/smll.202511992)
Supplement: Supplementary file 1 — Supporting File 1: smll72788‐sup‐0001‐SuppMat‐part1.pdf. [file SMLL-22-e11992-s001.pdf]

Polarization-dependent elliptical and rectangular Mie voids - Supplementary information

Serkan Arslan,[†,‡] Shaban B. Sulejman,[¶,‡] Sebastian Klein,[†] Jonathan Haehner,[§] Julian Schwab,[†] Dominik Ludescher,[†] Lukas Wesemann,[¶] Ann Roberts,[¶] Harald Giessen,[†] and Mario Hentschel[*,†]

†*University of Stuttgart, 4th Physics Institute, Germany*

‡*These authors contributed equally to this work*

¶*ARC Centre of Excellence for Transformative Meta-Optical Systems, School of Physics, The University of Melbourne, Victoria 3010, Australia*

§*School of Physics, University of Melbourne, Parkville, Australia*

E-mail: m.hentschel@physik.uni-stuttgart.de

Table S1: Nominal design values of the void radii R_x and R_y (in nanometers) corresponding to the structures presented in Supplementary Figs. S1–S10. The table arrangement reflects the sample layout, and the numbering matches that of the spectra in the same figures. In each array, the voids are arranged in a square lattice with a periodicity of $3R_x$ in both the x and y directions. Due to the electron beam lithography and etching–based fabrication process, the fabricated voids are typically larger than the nominal design values, with the error increasing for larger etching depths. In the main manuscript, the void dimensions were individually characterized by scanning electron microscopy (SEM), whereas here nominal values are reported.

Nr.86<br>750,750	Nr.87<br>750,700	Nr.88<br>750,650	Nr.89<br>750,600	Nr.90<br>750,550	Nr.91<br>750,500	Nr.92<br>750,450	Nr.93<br>750,400	Nr.94<br>750,350	Nr.95<br>750,300	Nr.96<br>750,250	Nr.97<br>750,200	Nr.98<br>750,150	Nr.99<br>750,100
Nr.73<br>700,700	Nr.74<br>700,650	Nr.75<br>700,600	Nr.76<br>700,550	Nr.77<br>700,500	Nr.78<br>700,450	Nr.79<br>700,400	Nr.80<br>700,350	Nr.81<br>700,300	Nr.82<br>700,250	Nr.83<br>700,200	Nr.84<br>700,150	Nr.85<br>700,100	
Nr.61<br>650,650	Nr.62<br>650,600	Nr.63<br>650,550	Nr.64<br>650,500	Nr.65<br>650,450	Nr.66<br>650,400	Nr.67<br>650,350	Nr.68<br>650,300	Nr.69<br>650,250	Nr.70<br>650,200	Nr.71<br>650,150	Nr.72<br>650,100		
Nr.50<br>600,600	Nr.51<br>600,550	Nr.52<br>600,500	Nr.53<br>600,450	Nr.54<br>600,400	Nr.55<br>600,350	Nr.56<br>600,300	Nr.57<br>600,250	Nr.58<br>600,200	Nr.59<br>600,150	Nr.60<br>600,100			
Nr.40<br>550,550	Nr.41<br>550,500	Nr.42<br>550,450	Nr.43<br>550,400	Nr.44<br>550,350	Nr.45<br>550,300	Nr.46<br>550,250	Nr.47<br>550,200	Nr.48<br>550,150	Nr.49<br>550,100				
Nr.31<br>500,500	Nr.32<br>500,450	Nr.33<br>500,400	Nr.34<br>500,350	Nr.35<br>500,300	Nr.36<br>500,250	Nr.37<br>500,200	Nr.38<br>500,150	Nr.39<br>500,100					
Nr.23<br>450,450	Nr.24<br>450,400	Nr.25<br>450,350	Nr.26<br>450,300	Nr.27<br>450,250	Nr.28<br>450,200	Nr.29<br>450,150	Nr.30<br>450,100						
Nr.16<br>400,400	Nr.17<br>400,350	Nr.18<br>400,300	Nr.19<br>400,250	Nr.20<br>400,200	Nr.21<br>400,150	Nr.22<br>400,100							
Nr.10<br>350,350	Nr.11<br>350,300	Nr.12<br>350,250	Nr.13<br>350,200	Nr.14<br>350,150	Nr.15<br>350,100								
Nr.5<br>300,300	Nr.6<br>300,250	Nr.7<br>300,200	Nr.8<br>300,150	Nr.9<br>300,100									
Nr.1<br>250,250	Nr.2<br>250,200	Nr.3<br>250,150	Nr.4<br>250,100										

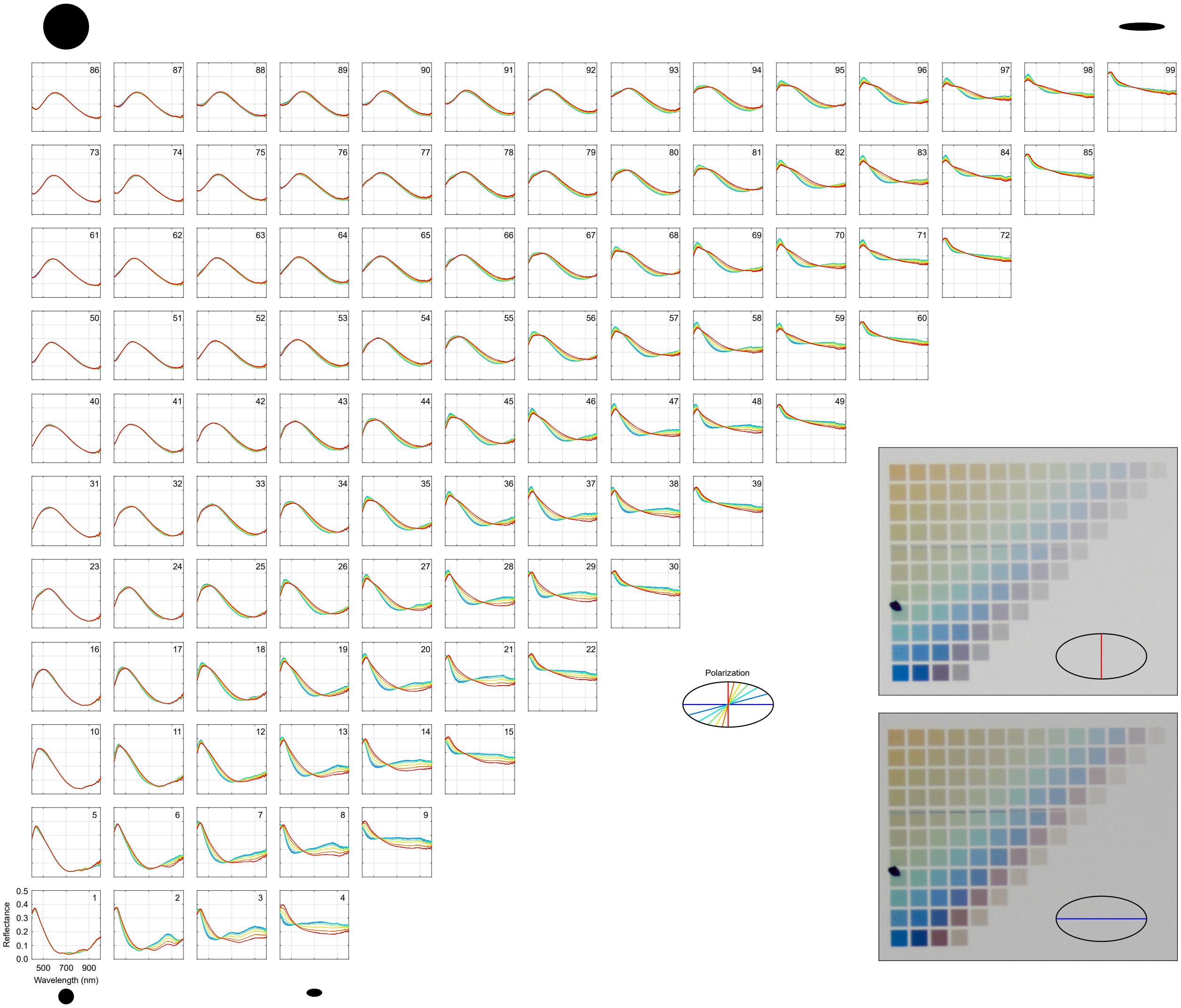

Figure S1: Two microscope images (bottom right) depict a parameter sweep of circular and elliptical Mie voids. The etching depth of the sample was approximately 290 nm, though the true depth of individual voids depends on their lateral dimensions. Specifically, larger voids tend to be shallower, while smaller voids are etched deeper due to an increased etching efficiency. Voids of a single size are arranged in square arrays with a side length of 25 µm. The nominal design values for R_x and R_y are listed in Table S1. Each row begins with a circular void on the left and moves towards a decreasing R_y from left to right while R_x remains fixed, thereby increasing the ellipticity. This leads to a stronger polarization dependence in the reflected colors, as visible by comparing the two microscope images (bottom right), which correspond to orthogonal polarization states. The void radii increase from bottom to top. The stylized circles/ellipses indicated in the corners of the figure illustrate the systematic parameter sweep in the size and aspect ratio. For each of the 99 arrays, the figure further shows the measured reflectance spectra in the wavelength range from 400-1000 nm. Seven polarization states were measured for each array, spanning from horizontal to vertical in 15° increments, as indicated by the color-coded ellipse in the center of the figure.

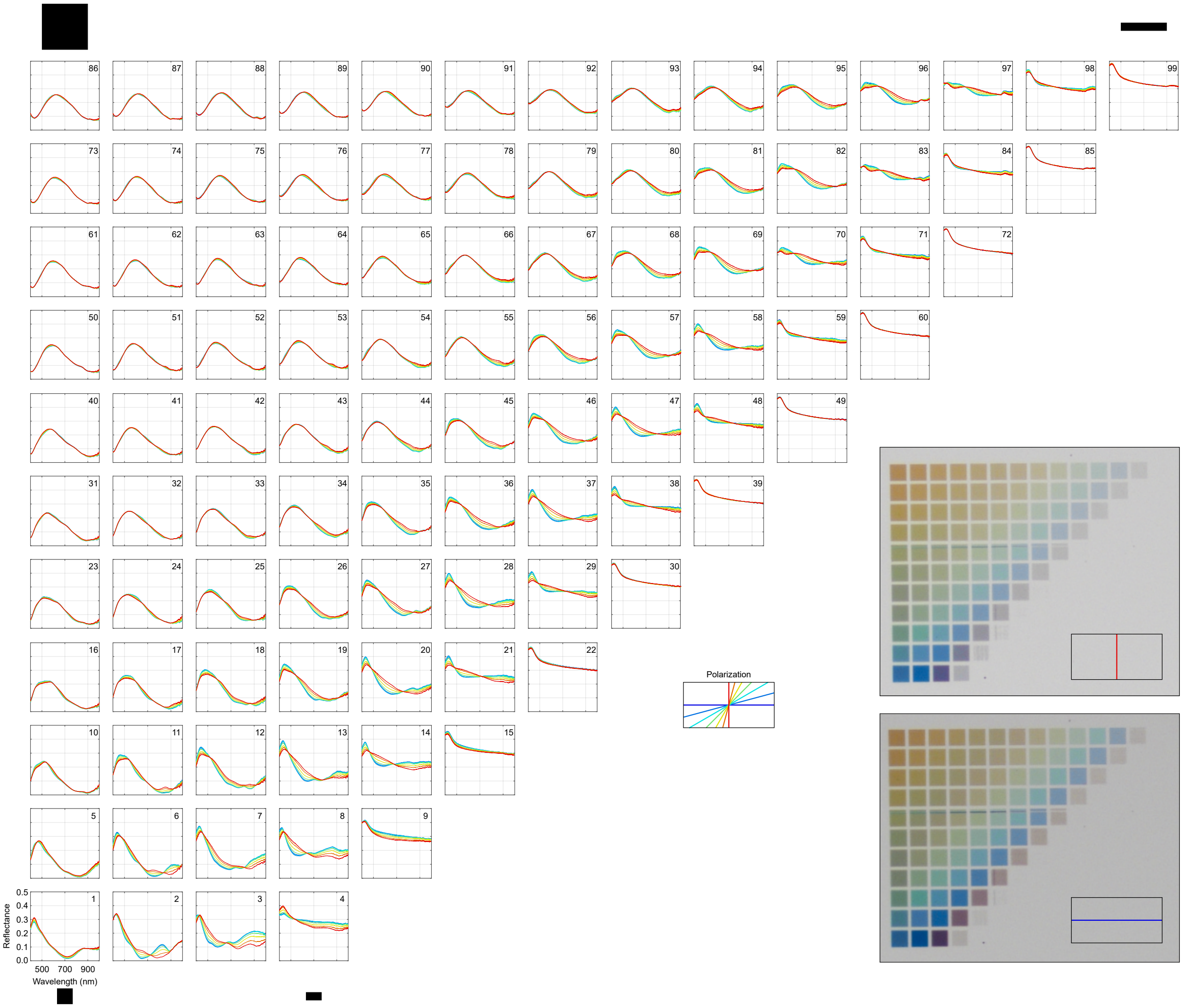

Figure S2: Two microscope images (bottom right) show a parameter sweep of square and rectangular Mie voids. The etching depth of the sample was approximately 290 nm. Voids of a single size are arranged in square arrays with a side length of 25 µm. The nominal design values for R_x and R_y are listed in Table S1. Each row begins with square voids on the left and moves toward a decreasing R_y from left to right while R_x remains fixed, thereby increasing the elongation. This leads to stronger polarization dependence in the reflected colors, as visible by comparing the two microscope images (bottom right), which correspond to orthogonal polarization states. From bottom to top, the void dimensions increase. The stylized squares and rectangles indicated in the corners of the figure illustrate the systematic sweep in the size and aspect ratio. For each of the 99 arrays, the figure further shows the measured reflectance spectra in the wavelength range from 400-1000 nm. Seven polarization states are measured for each array, spanning from horizontal to vertical in 15° increments, as indicated by the color-coded rectangle in the center of the figure.

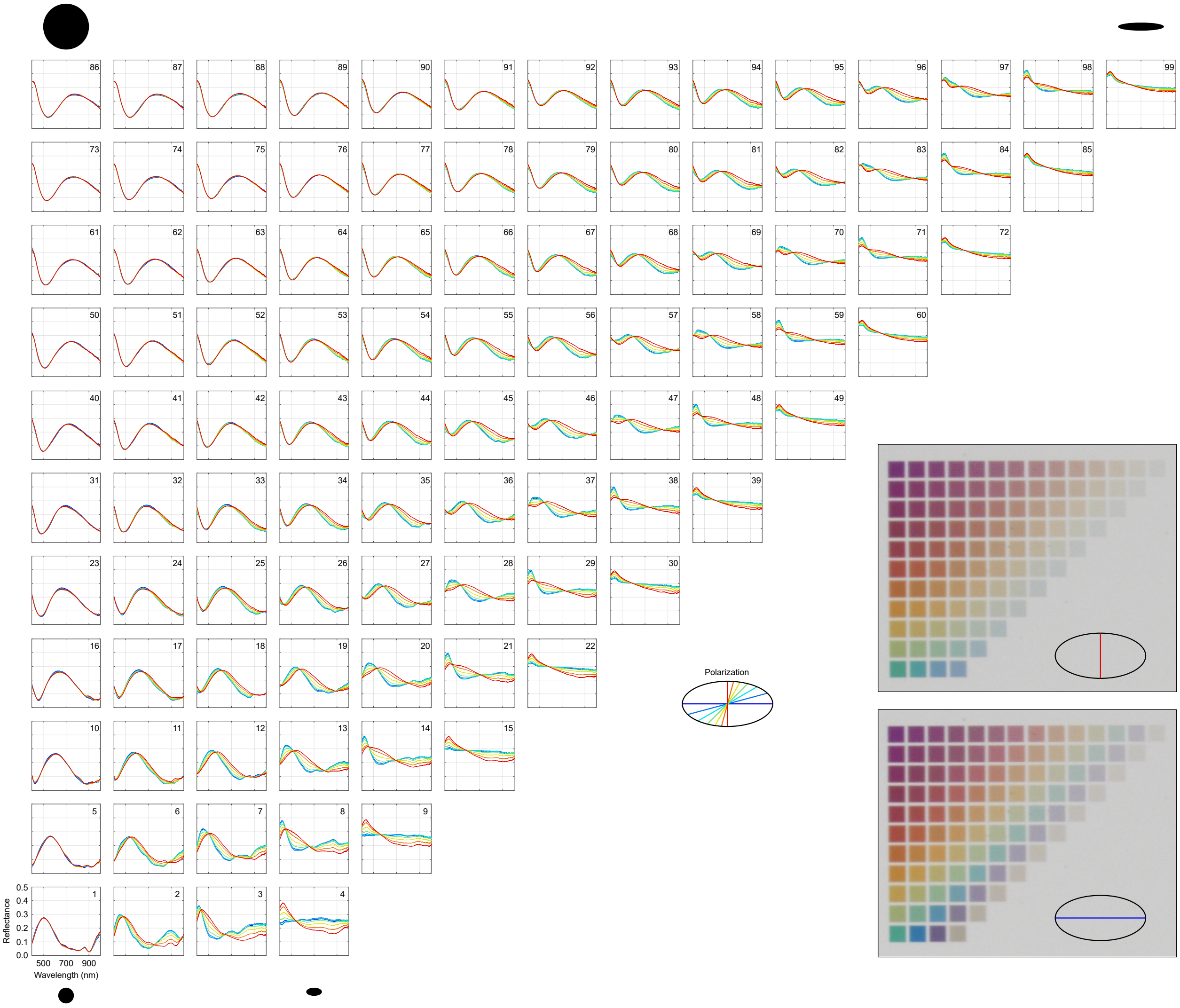

Figure S3: Two microscope images (bottom right) depict a parameter sweep of circular and elliptical Mie voids. The etching depth of the sample was approximately 380 nm and the voids of a single size were arranged in square arrays with a side length of 25 µm. The nominal design values for R_x and R_y are listed in Table S1. Each row begins with circular voids on the left. Moving from left to right, R_y decreases while R_x remains fixed, thereby increasing the ellipticity. This leads to a stronger polarization dependence in the reflected colors, as visible by comparing the two microscope images (bottom right), which correspond to orthogonal polarization states. From bottom to top, the void radii increases. The stylized circles/ellipses indicated in the corners of the figure illustrate the systematic sweep in the size and aspect ratio. For each of the 99 arrays, the figure further shows the measured reflectance spectra in the wavelength range from 400-1000 nm. Seven polarization states are measured for each array, spanning from horizontal to vertical in 15° increments, as indicated by the color-coded ellipse in the center of the figure.

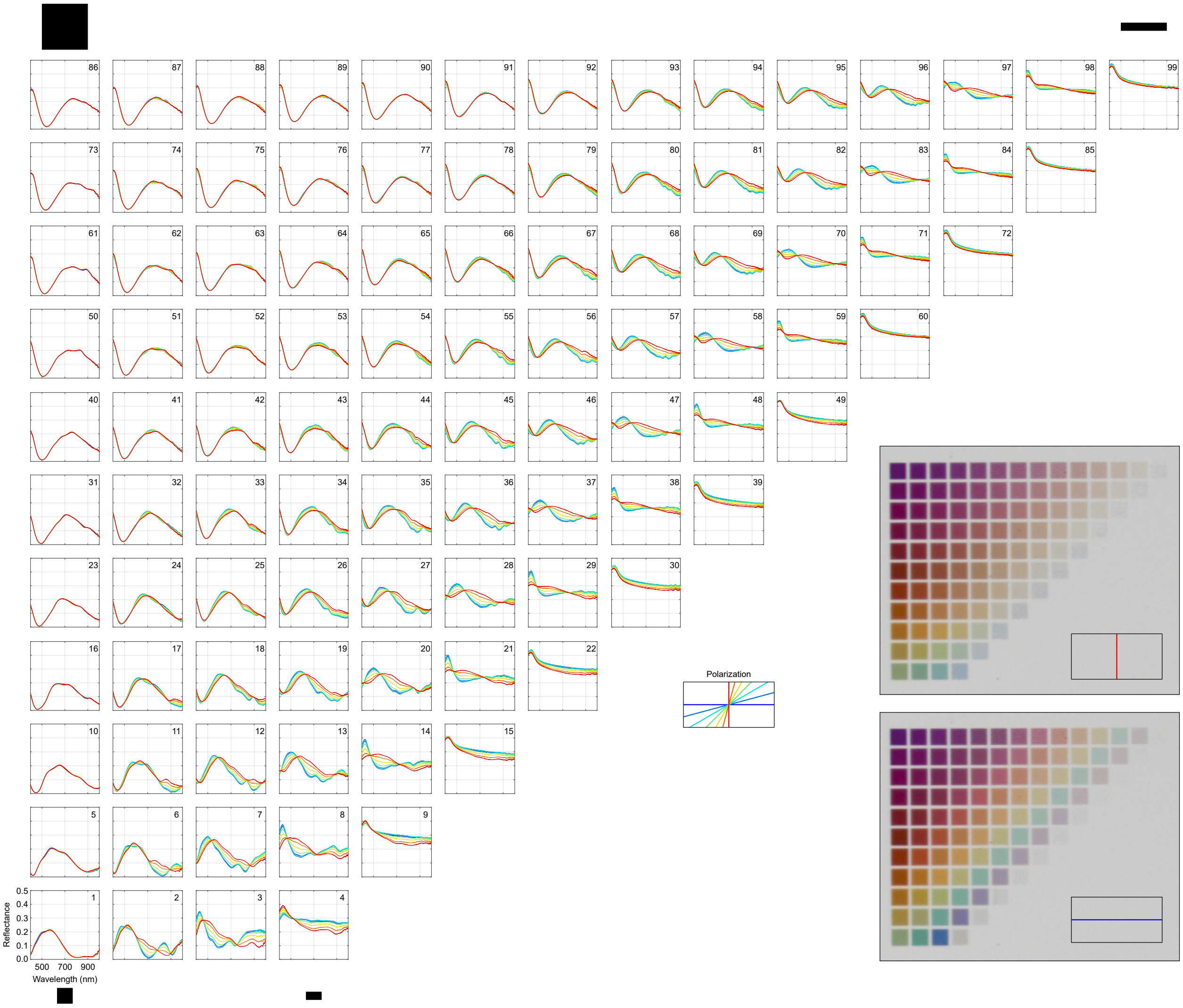

Figure S4: Two microscope images (bottom right) depict a parameter sweep of square and rectangular Mie voids. The etching depth of the sample was approximately 380 nm and voids of a single size were arranged in square arrays with a side length of 25 µm. The nominal design values for R_x and R_y are listed in Table S1. Each row begins with square voids on the left. Moving from left to right, R_y decreases while R_x remains fixed, thereby increasing the elongation. This leads to a stronger polarization dependence in the reflected colors, as visible by comparing the two microscope images (bottom right), which correspond to orthogonal polarization states. From bottom to top, the void dimensions increases. The stylized squares and rectangles indicated in the corners of the figure illustrate the systematic sweep in the size and aspect ratio. For each of the 99 arrays, the figure further shows the measured reflectance spectra in the wavelength range from 400-1000 nm. Seven polarization states are measured for each array, spanning from horizontal to vertical in 15° increments, as indicated by the color-coded rectangle in the center of the figure.

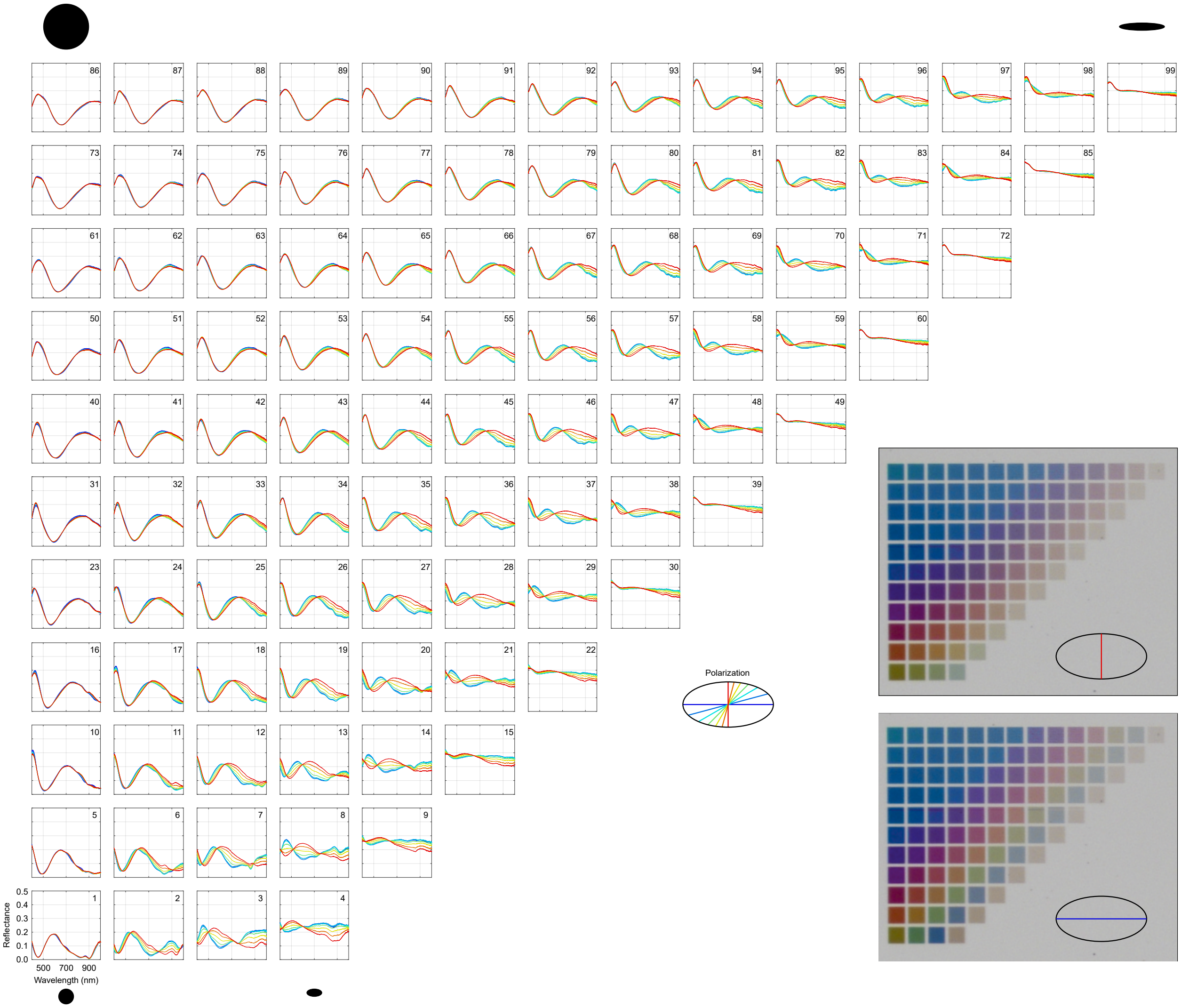

Figure S5: Two microscope images (bottom right) depict a parameter sweep of circular and elliptical Mie voids. The etching depth of the sample was approximately 480 nm and voids of a single size were arranged in square arrays with a side length of 25 µm. The nominal design values for R_x and R_y are listed in Table S1. Each row begins with circular voids on the left. Moving from left to right, R_y decreases while R_x remains fixed, thereby increasing the ellipticity. This leads to a stronger polarization dependence in the reflected colors, as visible by comparing the two microscope images (bottom right), which correspond to orthogonal polarization states. From bottom to top, the void radii increases. The stylized circles and ellipses indicated in the corners of the figure illustrate the systematic sweep in the size and aspect ratio. For each of the 99 arrays, the figure further shows the measured reflectance spectra in the wavelength range from 400-1000 nm. Seven polarization states are measured for each array, spanning from horizontal to vertical in 15° increments, as indicated by the color-coded ellipse in the center of the figure.

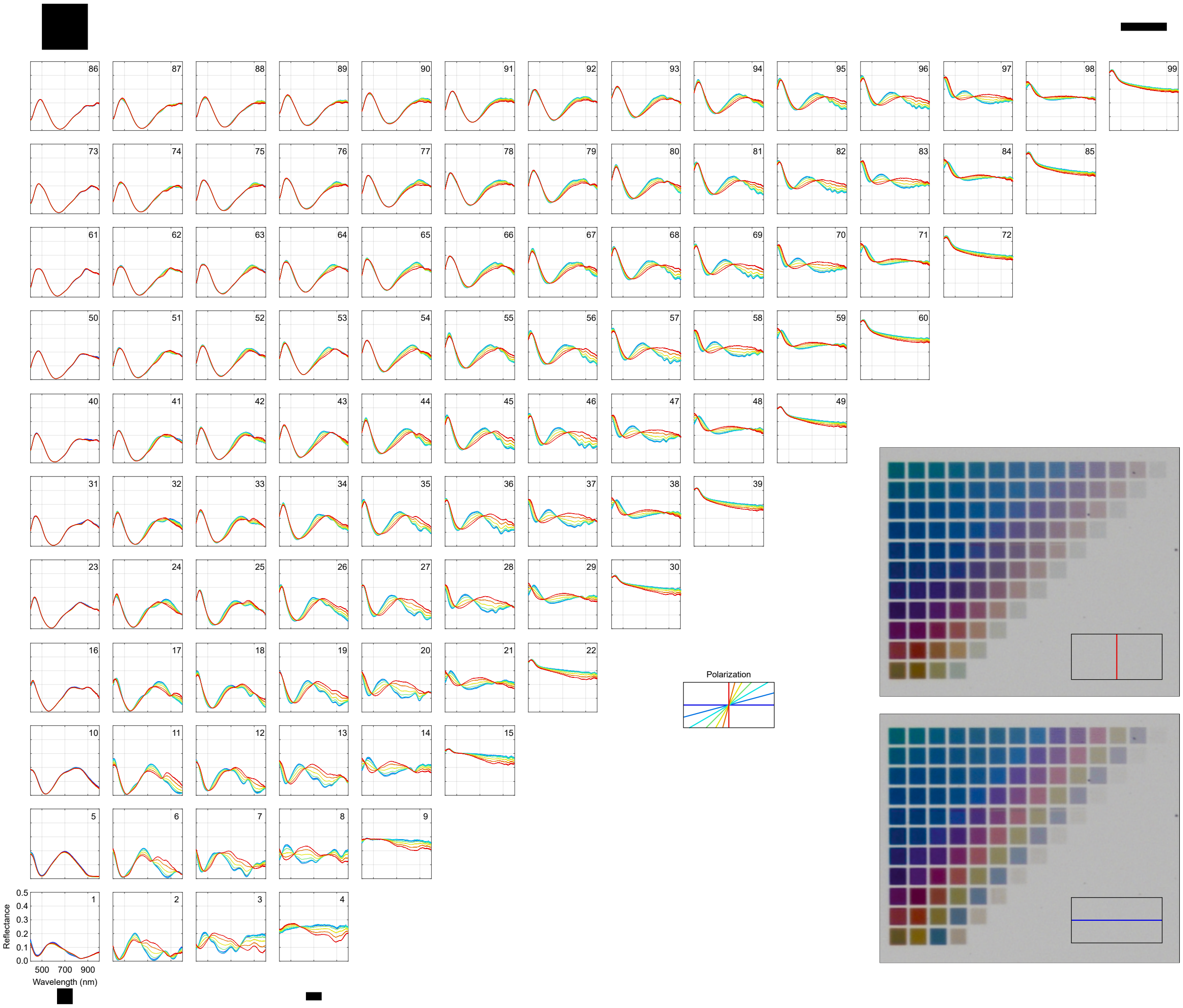

Figure S6: Two microscope images (bottom right) depict a parameter sweep of square and rectangular Mie voids. The etching depth of the sample is approximately 480 nm and voids of a single size are arranged in square arrays with a side length of 25 µm. The nominal design values for R_x and R_y are listed in Table S1. Each row begins with square voids on the left. Moving from left to right, R_y decreases while R_x remains fixed, thereby increasing the elongation. This leads to a stronger polarization dependence in the reflected colors, as visible by comparing the two microscope images (bottom right), which correspond to orthogonal polarization states. From bottom to top, the void dimensions increases. The stylized squares and rectangles indicated in the corners of the figure illustrate the systematic sweep in the size and aspect ratio. For each of the 99 arrays, the figure further shows the measured reflectance spectra in the wavelength range from 400-1000 nm. Seven polarization states are measured for each array, spanning from horizontal to vertical in 15° increments, as indicated by the color-coded rectangle in the center of the figure.
