# Supplementary material for: Polarization‐Dependent Elliptical and Rectangular Mie Voids
Source: Small. 2026 Feb 15;22(21):e11992. doi: 10.1002/smll.202511992 (PMC13081118; doi:10.1002/smll.202511992)
Supplement: Supplementary file 2 — Supporting File 2: smll72788‐sup‐0002‐SuppMat‐part2.pdf. [file SMLL-22-e11992-s002.pdf]

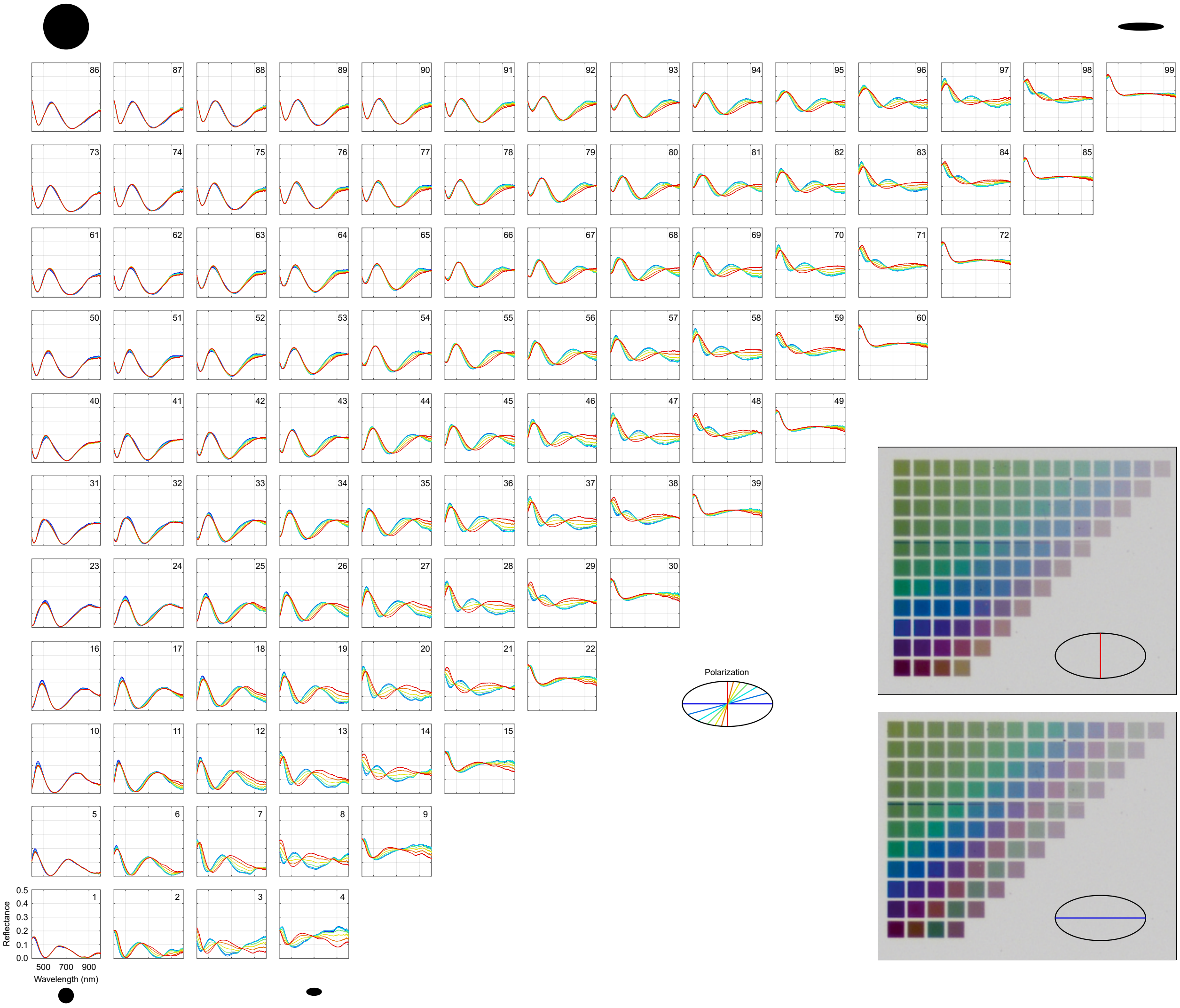

Figure S7: Two microscope images (bottom right) depict a parameter sweep of circular and elliptical Mie voids. The etching depth of the sample was approximately 560 nm and voids of a single size were arranged in square arrays with a side length of 25 µm. The nominal design values for R_x and R_y are listed in Table S1. Each row begins with circular voids on the left. Moving from left to right, R_y decreases while R_x remains fixed, thereby increasing the ellipticity. This leads to stronger polarization dependence in the reflected colors, as visible by comparing the two microscope images (bottom right), which correspond to orthogonal polarization states. From bottom to top, the void radii increases. The stylized circles and ellipses indicated in the corners of the figure illustrate the systematic sweep in the size and aspect ratio. For each of the 99 arrays, the figure further shows the measured reflectance spectra in the wavelength range from 400-1000 nm. Seven polarization states are measured for each array, spanning from horizontal to vertical in 15° increments, as indicated by the color-coded ellipse in the center of the figure.

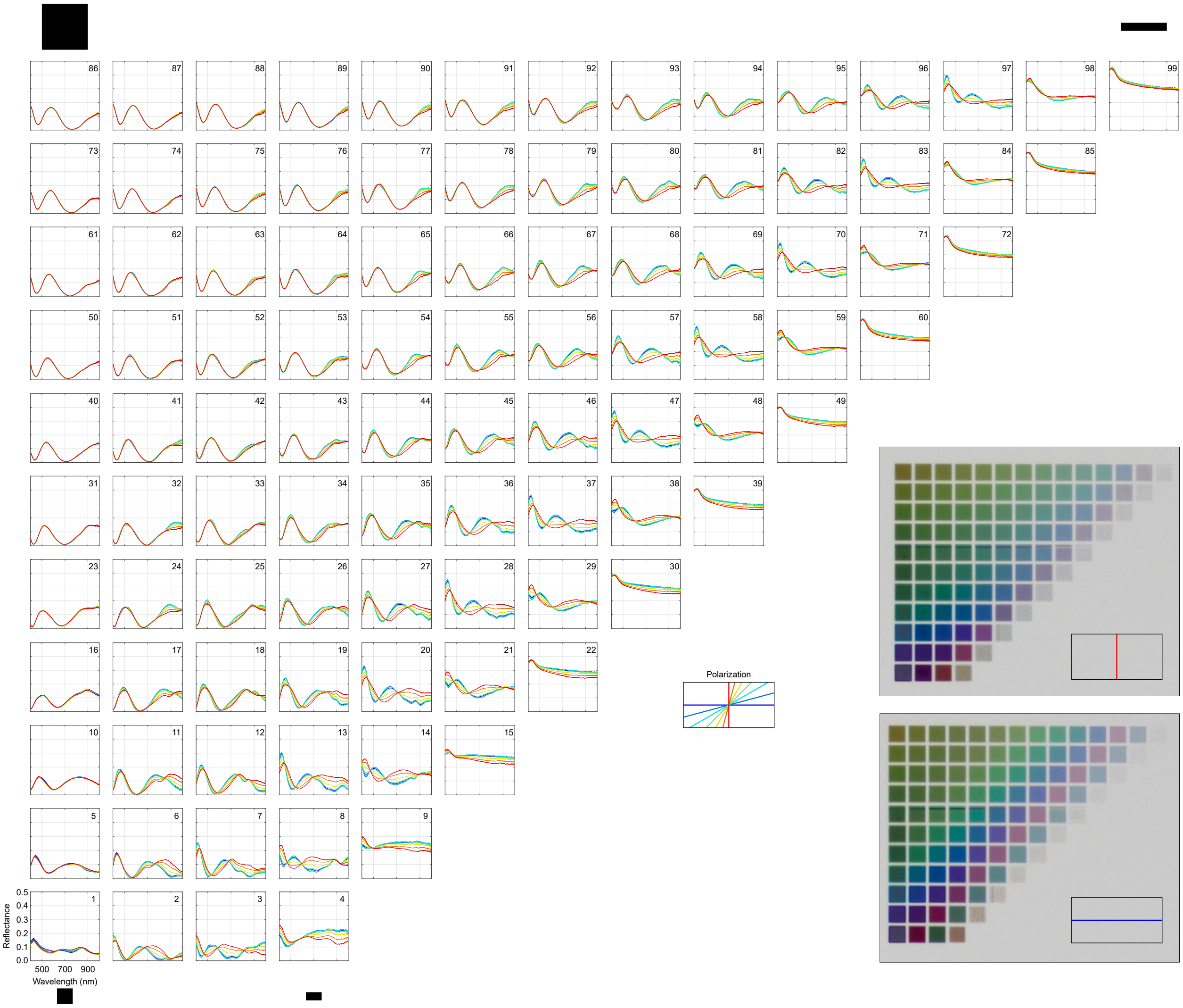

Figure S8: Two microscope images (bottom right) depict a parameter sweep of square and rectangular Mie voids. The etching depth of the sample was approximately 560 nm and voids of a single size were arranged in square arrays with a side length of 25 µm. The nominal design values for R_x and R_y are listed in Table S1. Each row begins with quadratic voids on the left. Moving from left to right, R_y decreases while R_x remains fixed, thereby increasing the elongation. This leads to a stronger polarization dependence in the reflected colors, as visible by comparing the two microscope images (bottom right), which correspond to orthogonal polarization states. From bottom to top, the void dimensions increases. The stylized squares and rectangles indicated in the corners of the figure illustrate the systematic sweep in the size and aspect ratio. For each of the 99 arrays, the figure further shows the measured reflectance spectra in the wavelength range from 400-1000 nm. Seven polarization states are measured for each array, spanning from horizontal to vertical in 15° increments, as indicated by the color-coded rectangle in the center of the figure.

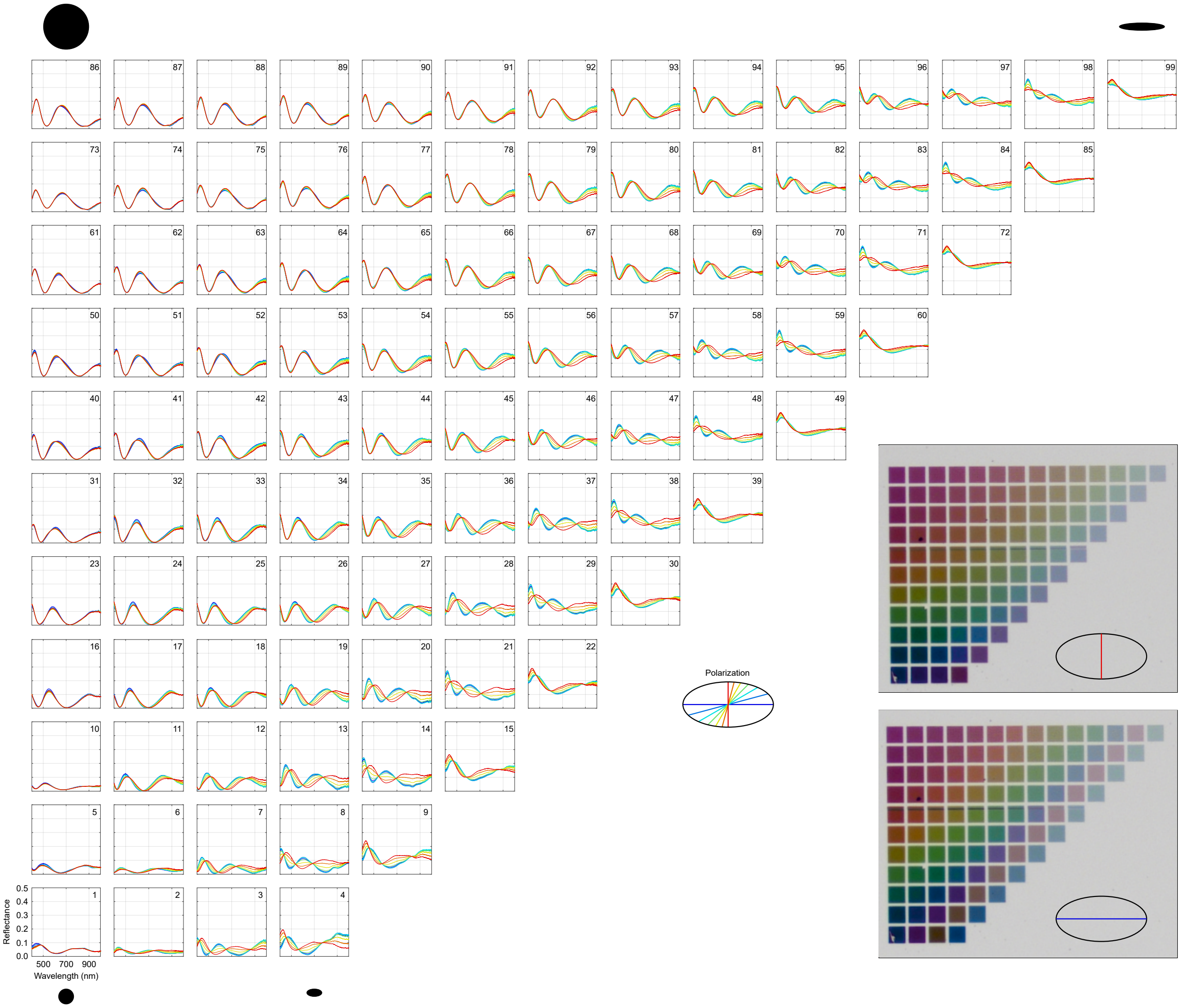

Figure S9: Two microscope images (bottom right) depict a parameter sweep of circular and elliptical Mie voids. The etching depth of the sample was approximately 630 nm and voids of a single size were arranged in square arrays with a side length of 25 µm. The nominal design values for R_x and R_y are listed in Table S1. Each row begins with circular voids on the left. Moving from left to right, R_y decreases while R_x remains fixed, thereby increasing the ellipticity. This leads to a stronger polarization dependence in the reflected colors, as visible by comparing the two microscope images (bottom right), which correspond to orthogonal polarization states. From bottom to top, the void radii increases. The stylized circles and ellipses indicated in the corners of the figure illustrate the systematic sweep in the size and aspect ratio. For each of the 99 arrays, the figure further shows the measured reflectance spectra in the wavelength range from 400-1000 nm. Seven polarization states are measured for each array, spanning from horizontal to vertical in 15° increments, as indicated by the color-coded ellipse in the center of the figure.

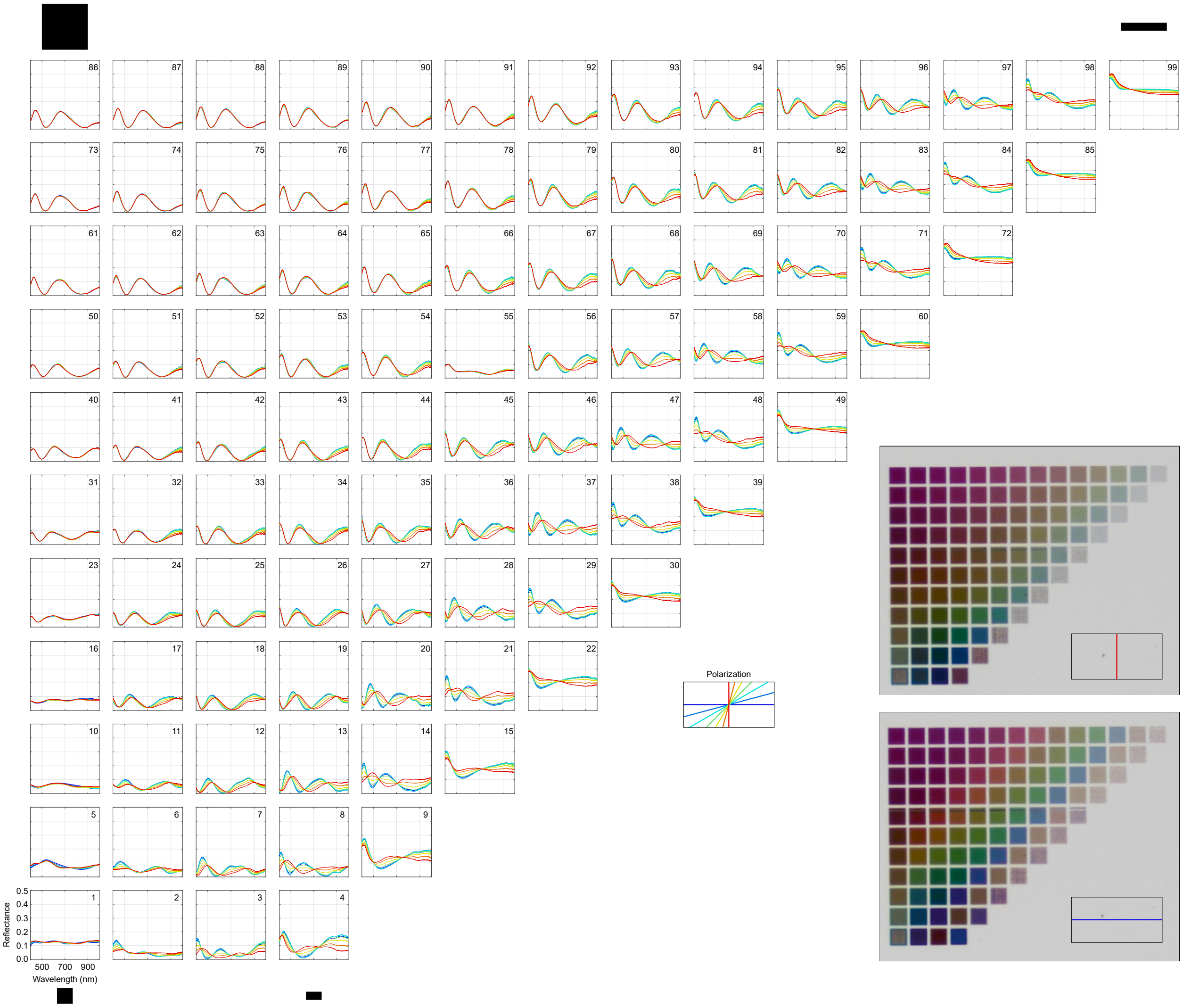

Figure S10: Two microscope images (bottom right) depict a parameter sweep of square and rectangular Mie voids. The etching depth of the sample was approximately 630 nm and voids of a single size were arranged in square arrays with a side length of 25 µm. The nominal design values for R_x and R_y are listed in Table S1. Each row begins with square voids on the left. Moving from left to right, R_y decreases while R_x remains fixed, thereby increasing the elongation. This leads to a stronger polarization dependence in the reflected colors, as visible by comparing the two microscope images (bottom right), which correspond to orthogonal polarization states. From bottom to top, the void dimensions increases. The stylized squares and rectangles indicated in the corners of the figure illustrate the systematic sweep in the size and aspect ratio. For each of the 99 arrays, the figure further shows the measured reflectance spectra in the wavelength range from 400-1000 nm. Seven polarization states are measured for each array, spanning from horizontal to vertical in 15° increments, as indicated by the color-coded rectangle in the center of the figure.

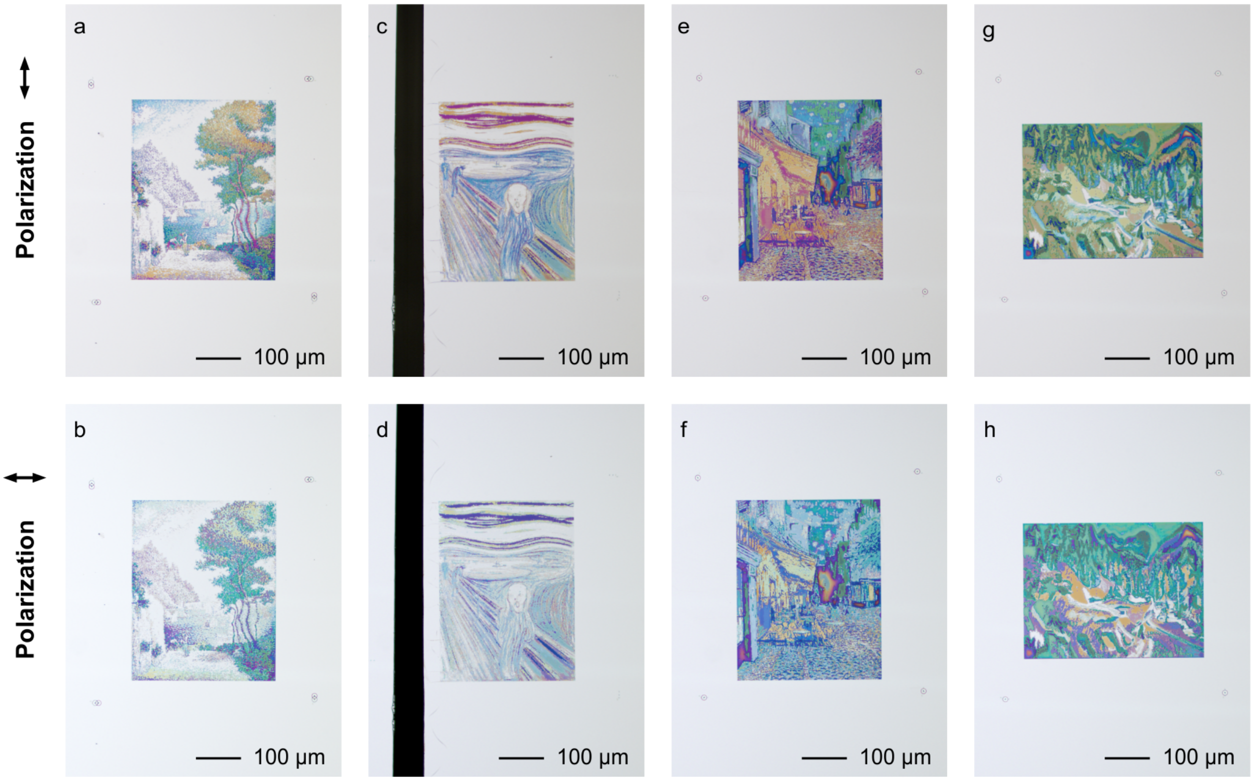

Figure S11: Microscope images of four nanofabricated color prints under vertical (top) and horizontal (bottom) polarization. As the structures were designed for only a specific polarization state, the images in the top row show the desired color impressions, while the hues in the bottom row images appear weakened. The original painting names, year of creation and artist from left to right are: *Capo di Noli* (1898) by Paul Signac, reproduced with permission (Photocredit "Capo di Noli" by "Paul Signac", photocopyright "Wallraf-Richartz Museum & Fondation Corboud, Köln, Germany"), *The Scream* (1893) by Edvard Munch, reproduced with permission (Photocredit "The Scream" by "Edvard Munch", photocopyright "National Museum of Art, Architecture and Design, Oslo, Norway"), *Café Terrace at Night* (1888) by Vincent van Gogh, reproduced with permission (Photocredit "Café Terrace at Night" by "Vincent van Gogh", photocopyright "Kröller Müller Museum, Otterlo, Netherlands"), and *Landschaft Sertigtal* (1924) by Ernst Ludwig Kirchner, reproduced with permission (Photocredit "Landschaft Sertigtal" by "Ernst Ludwig Kirchner", photocopyright "Collection of Gabriele and Werner Merzbacher, Kunsthaus Zürich, Zürich, Switzerland").

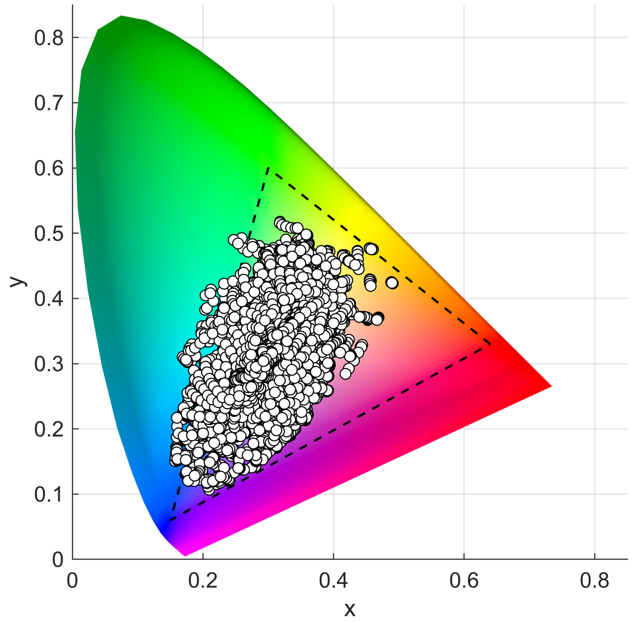

Figure S12: The CIE (1931) diagram depicts the wide color gamut achieved with the Mie void pallet presented in the supplementary figures above, which forms the basis for the nanoscale color printing presented in Figure 5 of the manuscript as well as S11. The dotted triangle indicates the sRGB color space.
